# Differential reproductive plasticity under thermal variability in a freshwater fish (*Danio rerio*)

**DOI:** 10.1098/rspb.2022.0751

**Published:** 2022-09-14

**Authors:** Melanie D. Massey, M. Kate Fredericks, David Malloy, Suchinta Arif, Jeffrey A. Hutchings

**Affiliations:** ^1^ Department of Biology, Life Sciences Centre, Dalhousie University, 6299 South St, Halifax, NS, Canada B3H 4R2; ^2^ Zebrafish Core Facility, Dalhousie University, Halifax, Nova Scotia, Canada; ^3^ Flødevigen Marine Research Station, Institute of Marine Research, Bergen, Norway; ^4^ Department of Natural Sciences, University of Agder, Kristiansand, Norway

**Keywords:** acclimation, developmental plasticity, fecundity, reproduction, thermal variability, thermal fluctuations

## Abstract

Human-driven increases in global mean temperatures are associated with concomitant increases in thermal variability. Yet, few studies have explored the impacts of thermal variability on fitness-related traits, limiting our ability to predict how organisms will respond to dynamic thermal changes. Among the myriad organismal responses to thermal variability, one of the most proximate to fitness—and, thus, a population's ability to persist—is reproduction. Here, we examine how a model freshwater fish (*Danio rerio*) responds to diel thermal fluctuations that span the species's viable developmental range of temperatures. We specifically investigate reproductive performance metrics including spawning success, fecundity, egg provisioning and sperm concentration. Notably, we apply thermal variability treatments during two ontogenetic timepoints to disentangle the relative effects of developmental plasticity and reversible acclimation. We found evidence of direct, negative effects of thermal variability during later ontogenetic stages on reproductive performance metrics. We also found complex interactive effects of early and late-life exposure to thermal variability, with evidence of beneficial acclimation of spawning success and modification of the relationship between fecundity and egg provisioning. Our findings illuminate the plastic life-history modifications that fish may undergo as their thermal environments become increasingly variable.

## 1. Background

Phenotypic plasticity is a universal property of living organisms, facilitating both acute and long-term nongenetic responses to changes in environmental conditions [1–3]. The nature of plastic responses is complex and dependent on numerous factors, including the species and phenotypic traits under study, as well as the ontogenetic timing and duration of exposure to environmental signals [3]. As organisms undergo early development, they may alter their ontogenetic trajectories through developmental plasticity, a process which is often considered irreversible [2]. Throughout their lifetimes, organisms may also mount reversible responses to environmental inputs through the process of acclimation [4]. Moreover, developmental plasticity and acclimation are mechanistically linked and can interact to produce a range of phenotypic responses [5]. An existing goal of physiologists is to disentangle and describe the relative and interactive contributions of these two forms of plasticity, especially given recent suggestions that plasticity will play a critical role in mediating the biological impacts of climate change [6,7].

A growing body of literature has examined the differential and interactive effects of developmental plasticity and acclimation in a variety of traits, particularly in the context of thermal acclimation. Many of these studies sought to explore whether early exposure to one environment resulted in improved performance under those conditions later in life, as tests of the beneficial acclimation hypothesis (BAH) [8] in the broadest sense of the term [4,9–11]. For example, both developmental and adult acclimation of fruit flies to cool temperatures result in increased cold tolerance during assays later in life [12], and female seed beetles display higher fitness when developmental and adult reproductive temperatures match [10]. Furthermore, recent studies in fish species have suggested that beneficial developmental acclimation of metabolism [13,14] and reproduction [15,16] can occur in response to warm temperatures. With that said, examples of beneficial acclimation tend to be the exception rather than the rule [4]; more often than not, there appears to be a developmentally optimum environment which results in the strongest performance later in life across a range of adult conditions [4,9,17]. Moreover, authors have occasionally detected opposing responses of developmental plasticity and acclimation to the same environmental conditions [9,18], highlighting the importance of ontogenetic timing of exposure to environmental conditions. Taken together, these equivocal results suggest that developmental plasticity, acclimation and their interaction may significantly affect phenotypic outcomes, but that the nature of these outcomes can be context-dependent and unpredictable.

Another major challenge of generalizing conclusions across acclimation experiments is that the environmental treatments used often do not reflect ecologically realistic conditions. In studies of thermal plasticity, much of what is currently known is derived from studies that employ simplified constant temperature regimes [19] (but see [20]). Although constant temperature experiments have generated remarkable and seminal biological insights [19], they probably reflect evolutionarily novel environments, and their applicability to natural conditions are limited [21,22]. It has also been suggested that stressful static conditions commonly chosen to examine the effects of temperature may themselves impose detrimental pathologies, obfuscating the consequences of thermal acclimation [4,23]. For this reason, authors have advocated for the use of thermal variability over constant temperature conditions, to mitigate the confounding effect imparted by stressful or ecologically irrelevant temperatures [4,23].

Indeed, there are few studies that leverage thermal variability to investigate the contributions of different forms of plasticity to phenotypic variation [24,25]. Schaefer & Ryan [24] determined that developmental plasticity, acclimation, and their additive interaction in response to a broad range of diel fluctuating temperatures significantly increased the heat tolerance of zebrafish (*Danio rerio*). Bilcke, Downes & Büscher [25] likewise found evidence of developmental beneficial acclimation of locomotor and predation performance in common garden skinks using ecologically realistic treatment temperatures. These studies suggest that there may be appreciable plastic responses to thermal variability, and in some cases, these responses impart important benefits to organismal performance.

In the present study, we estimate the relative and combined influences of developmental plasticity under thermal variability, comparing these effects with constant optimal temperatures. We implement a factorial experimental approach (*sensu* [26]), applying thermal treatments during early ontogeny (embryonic and larval stages) and late ontogeny (juvenile and adult stages) in zebrafish, a model organism. Specifically, we measure key elements of reproductive performance, including spawning success, fecundity and egg provisioning and sperm quality. We chose reproductive performance because it is strongly influenced by temperature-mediated plasticity in ectotherms [6,27–31], and fitness correlates represent the most pertinent metrics for investigating the importance of plasticity [4,10,23].

Here, we test for evidence of beneficial acclimation of reproductive traits, which we describe as a positive interaction between early and late ontogenetic environments, such that fish exposed to thermal variability during early life may perform better under those same conditions later in life. We consider this an extension of the BAH [10,11,26]. Aside from the BAH, we also examine both positive and negative responses to thermal variability, teasing apart the contributions of developmental plasticity and acclimation. Last, because fecundity and egg allocation are correlated through trade-offs in a life-history framework [32], we examine their plastic responses jointly to test whether life history can be modified by thermal variability [10].

## 2. Methods

### (a) Parental fish rearing and breeding

We initiated our experiment in February 2021 with 600 freshly laid (less than 4 h post-fertilization) F_0_ zebrafish eggs from an ancestral stock of three wild-type AB lineages acquired from the Dalhousie University Zebrafish Core Facility (ZCF). For a detailed explanation of rearing conditions, see electronic supplemental material.

### (b) Thermal treatments during ‘early’ and ‘late’ ontogeny

**Figure 1.**
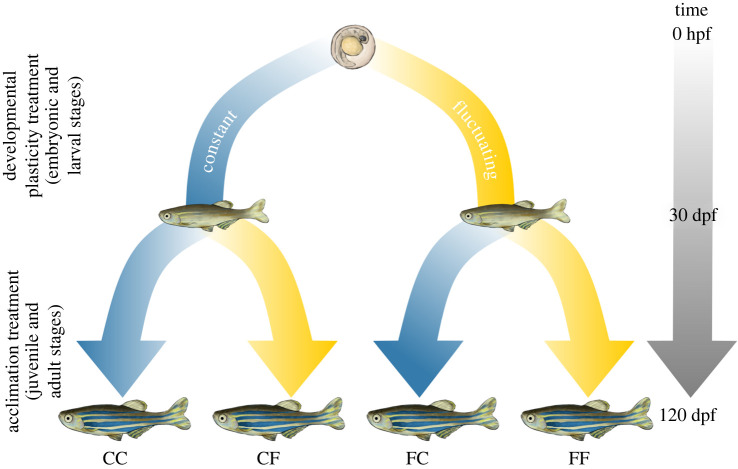
Flowchart illustrating split-clutch experimental design. Freshly laid zebrafish (*Danio rerio*) eggs were split into constant (C; 27°C) or fluctuating (F; 22–32°C, on a diel cycle) treatment groups for embryonic and larval development, producing a developmental plasticity treatment (0 to 30 dpf). At the onset of the juvenile stage, they were split once more into constant and fluctuating treatment groups, resulting in an acclimation treatment (30–120 + dpf). Ultimately, this full-factorial design created four groups with combinations of developmental plasticity and acclimation treatments: constant–constant (CC), constant–fluctuating (CF), fluctuating–constant (FC) and fluctuating–fluctuating (FF). hours post-fertilization (hpf), days post-fertilization (dpf). (Online version in colour.)

We manipulated temperature during ‘early’ (embryonic and larval stages; 0–29 days post-fertilization) and ‘late’ (juvenile and adult stages; 30+days post-fertilization) ontogeny of F_0_ fish, employing a full-factorial, split-clutch design (figure 1). The early period ultimately represented the developmental plasticity treatment, whereas the late period represented the acclimation treatment.

We randomly and evenly split clutches of freshly laid eggs from three lineages (pairings) of F_0_ zebrafish into Constant or Fluctuating treatments, representing the developmental plasticity phase. Fish remained in these treatments until the onset of the Late ontogenetic period, then we once again split groups into constant and fluctuating treatments, representing the acclimation phase. This factorial design resulted in four groups that experienced a combination of early and late thermal treatments (figure 1): constant–constant (CC), constant–fluctuating (CF), fluctuating–constant (FC) and fluctuating–fluctuating (FF).

We selected 27°C as our constant temperature treatment and a diel fluctuation from 22–32°C as our fluctuating treatment. Warm temperatures were set to peak at 12.00 p.m., and cool temperatures were set to peak at 12.00 a.m. These thermal treatments were designed to have different magnitudes of variability while maintaining an equal thermal mean (approx. 27°C). Whereas temperatures ranging from 26–28°C are often considered constant ‘optimal’ temperatures for laboratory zebrafish (e.g. promoting growth, fecundity, and immune responses [33,34]), 22–32°C represents the maximum range of temperatures under which zebrafish develop normally, representing the extreme developmental thermal boundary beyond which high levels of mortality, deformation and thermal stress occur [35,36]. Yet, during reproductive season in natural habitats, temperatures tend to vary from approximately 23–31°C [34]. As such, the Fluctuating regime represents a physiologically challenging [35] but ecologically realistic range of temperatures. Our temperature treatment system is described in electronic supplemental material.

### (c) Size and spawning success in fish exposed to thermal treatments during ‘early’ and ‘late’ ontogeny

In May 2021, when fish were sexually mature (120 d old), we began breeding experiments. Zebrafish are seasonal batch-spawners, with the ability to spawn continuously after reaching sexual maturity [34], though reproductive effort is largely expended during monsoon season, characterized by high environmental variability, in their natural habitats [37]. Further, in the wild, they generally exhibit an annual life cycle, typically experiencing only one reproductive season [34]. To reflect natural conditions, we conducted breedings once per week over the course of five weeks for each lineage to attain an estimate of female fecundity. A one-week rest period between spawnings has been shown to be sufficient to allow zebrafish to recuperate their reproductive investment [38].

To measure spawning success, which we define as the production of any eggs by a breeding pair, we randomly selected pairs of sibling males and females from the same treatment tank. Sibling pairs, rather than between-family crosses, were selected so that we could later delineate family-level effects from treatment effects. We placed breeding pairs in a zebrafish breeding box connected to the flow-through system the afternoon before breeding. We separated males and females using a clear plastic divider and placed identical sterilized plastic plants in each female's compartment to stimulate egg production [39]. The next morning, immediately after the onset of the light period at 08:00 h, we disconnected the flow-through system from breeding tanks and removed dividers. We elevated one end of each tank by 5 cm to create a gradient of water depth to stimulate breeding, and allowed pairs to breed for 3 h. After this period, fish were sedated via inhalation of buffered MS-222 (80 mg l^−1^), weighed, and measured for standard length (SL) as our metric of body size, before being returned to their tank of origin.

### (d) Female reproductive traits: egg counts and measurements

Female zebrafish will occasionally produce necrotic, ‘non-viable’ eggs, as the result of resorption of mature ova; these non-viable eggs are identified by an opaque and asymmetric appearance [34]. We collected and sorted F_1_ eggs from each spawning event, separating out non-viable eggs. We placed viable eggs in petri dishes filled with E3 embryo medium, and photographed them under a dissecting microscope, using a 0.001 cm micrometer for size calibration. We took the production of any eggs (viable or non-viable) to indicate that spawning took place (i.e. breeding conditions stimulated the female to produce eggs), but only included counts of viable eggs in our estimates of fecundity.

To estimate egg provisioning, we measured equatorial yolk diameter for a random subsample of up to 10 viable eggs per spawning using ImageJ (National Institutes of Health, Bethesda, MD). Yolk volume is a common and suitable proxy for maternal provisioning of eggs [40], and is relevant given its correlation with offspring fitness in oviparous ectotherms [41].

### (e) Male reproductive traits: sperm concentration

Sperm concentration and volume are both significantly correlated with sperm quality in zebrafish, and are positively associated with higher rates of fertilization and lower rates of offspring deformity [42]. We thus used sperm concentration as a proxy for male reproductive quality. In September 2021, two weeks after the last breeding, we measured sperm concentration and volume from 8–12 randomly selected F_0_ males per treatment. We collected and measured sperm from anesthetized males taken directly from home tanks, using 10 µl glass microcapillary tubes. A visual assessment of the collected sperm was then made to minimize urine or faeces contamination in samples [43]; poor quality samples indicated by low opacity or fecal content were removed from analyses. Known volumes of sperm from each male were then diluted with 4 µl of E400 medium. The absorbance of the resulting sample was measured at 400 nm using a Nanodrop Spectrophotometer (Thermo Fisher Scientific, Waltham, MA), and sperm concentration (sperm cells/mL) was estimated by using a hemocytometer-calibrated standard curve [44].

### (f) Statistical analyses

We applied Bayesian linear mixed models to estimate ‘early’ and ‘late’ thermal treatment effects (two level factors) and their interaction on parental body sizes, spawning success, fecundity, and yolk volume. Additionally, we used a Bayesian linear model to estimate thermal treatment effects on sperm concentration. In all models we included 'family' (three-level, categorical) as a covariate to investigate the variability in effect sizes owing to family-level effects, with the exception of sperm concentration, in which males from different families were pooled due to small sample size. Finally, we included paternal size as a covariate for breeding success and sperm concentration models, as paternal size is expected to influence both metrics [45], and we sought to estimate direct effects of thermal treatments. Likewise, we included maternal size as a covariate for both fecundity and yolk volume models as female size is expected to influence both metrics [46], and to isolate direct treatment effects. Because measurements on individual tanks were repeated weekly over five weeks (for all metrics except sperm concentration), we included 'tank' as a random intercept term to account for repeated measures [47].

We used a binomial distribution to model breeding success, a negative binomial distribution with a log link function for fecundity (to account for overdispersion detected in pilot Poisson models [48]), and gaussian distributions with identity link functions for parental body size, yolk volume, variation in yolk volume, and sperm concentration models. We ran all models using the ‘rstanarm’ package in the R environment (4.1.0), using weakly informative default priors to provide moderate regularization [49]. Model fits were further validated through joint and pointwise (median and skew) posterior predictive checks to ensure real data fit reasonably within simulated model predictions. We also cross-validated models using Pareto-smoothed importance sampling (PSIS-LOO) cross-validation (electronic supplemental material).

We visually described model results using plots of posterior median values from Bayesian models, which are analogous to parameter estimates in frequentist models, and can be directly interpreted as ‘effect sizes’. These effect sizes intuitively represent the relative strength of each parameter's effect on response variables. We further included 50% and 90% uncertainty intervals (UIs), which illustrate certainty in parameter estimates based on the posterior generated from each model. UIs can be interpreted analogously to frequentist confidence intervals.

We used a Bayes factor analysis to estimate the relative likelihood of a difference between treatment groups (CC, CF, FC and FF). Briefly, Bayes factor (BF10) greater than 1 favours the hypothesis that two groups are different. Based on the framework laid out by [42], a BF10 of 1–3 indicates weak evidence, 3–20 indicates positive evidence, 20–150 indicates strong evidence and greater than 150 indicates very strong evidence of a difference between groups (see electronic supplementary material for details).

## 3. Results

### (a) Thermal treatments

**Figure 2.**
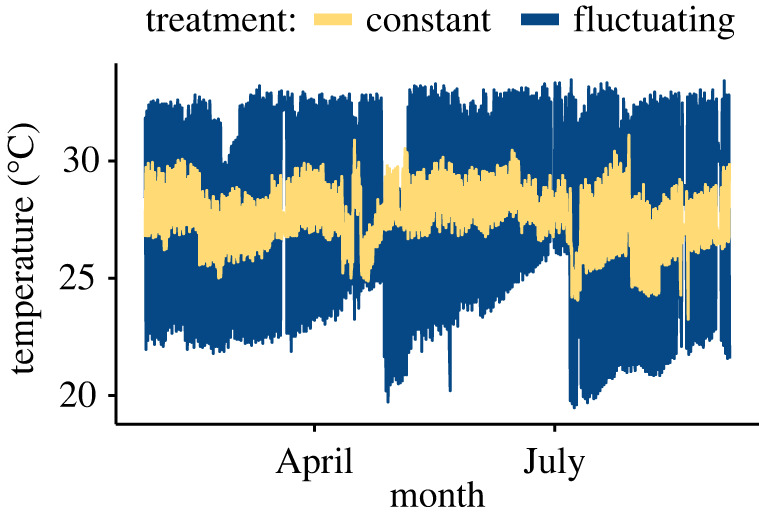
Temperature logs of thermal treatments from January to September 2021. The constant temperature treatment (yellow) is plotted in the foreground, and the diel fluctuating treatment (blue) lies behind; the two treatments were designed to share a common thermal mean. (Online version in colour.)

The mean temperature of the constant treatment throughout the experiment was 27.58 (±1.23 s.d.) °C, and the mean temperature of the fluctuating treatment was 28.18 (± 3.64 s.d.) °C (figure 2). The constant treatment was slightly more variable than anticipated due to constraints on our water supply system, and both treatments were subject to slight seasonal variation in ambient water temperatures provided to our facility.

### (b) Effect of thermal variability on male and female body sizes

**Figure 3.**
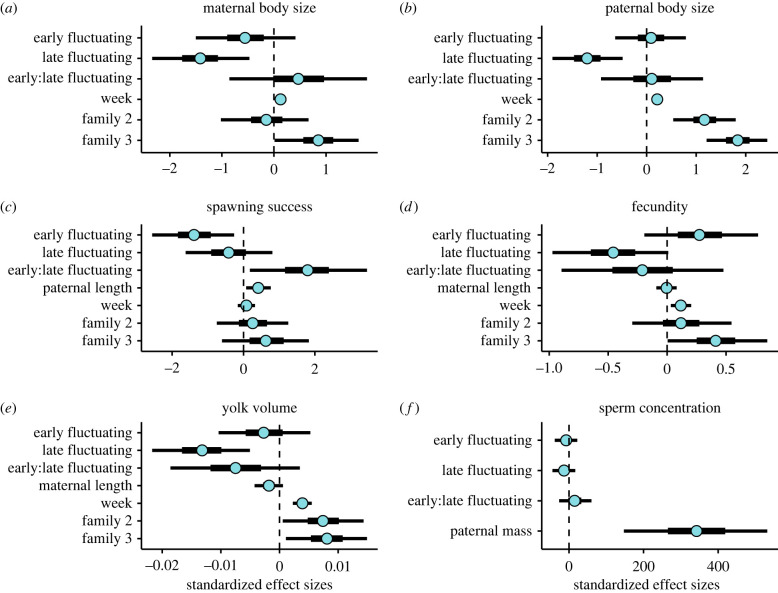
Standardized effect sizes (posterior medians) of covariates and predictors (*y*-axis labels) for Bayesian mixed models (*a–e*) and linear model (*f*) of body sizes and reproductive traits. Blue dots indicate effect sizes, thick black lines indicate 50% uncertainty intervals (UIs), and thin black lines indicate 90% UIs. Briefly, effect sizes that are further from 0 suggest stronger effects, and UIs that cross 0 suggest less certainty in estimates. (Online version in colour.)

**Figure 4.**
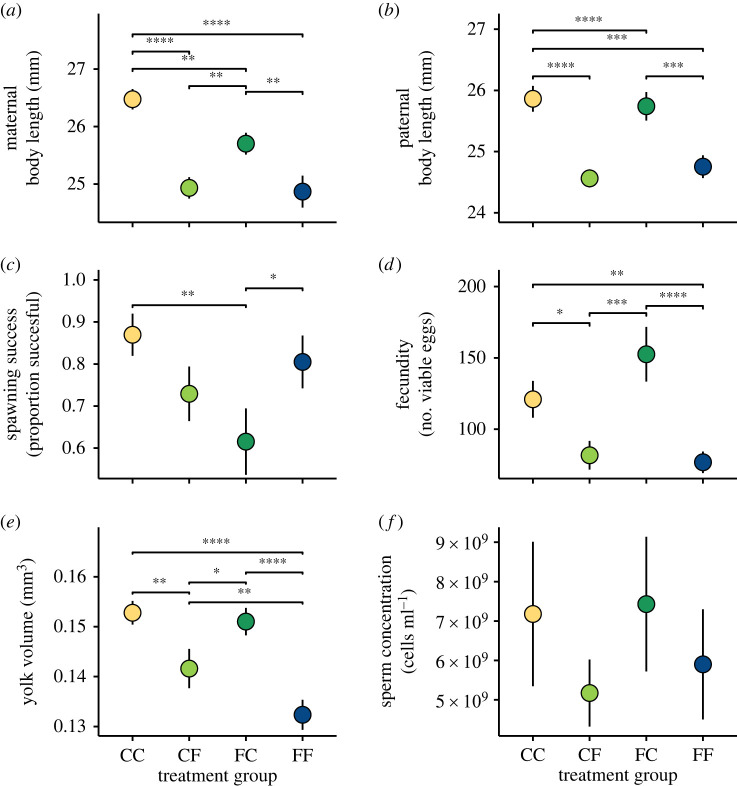
Means (±SE) of body size and reproductive traits (*a–f*) for each treatment group in the study. Lines above pairings indicate pairs for which there is evidence of a difference based on their paired Bayes factor (BF_10_). * indicates weak evidence, ** indicates positive evidence, *** indicates strong evidence, and **** indicates very strong evidence toward differences between groups. (Online version in colour.)

Based on the Bayesian mixed models for body sizes, there was a negative effect of early fluctuating temperature on maternal body size, with moderate certainty such that 95% uncertainty intervals (UIs) crossed zero, but 50% UIs did not (figure 3*a,b*). Late Fluctuating temperature exhibited a stronger negative effect on both maternal and paternal body sizes (figure 3*a,b*). Both maternal and paternal body sizes were influenced by family lineage (figure 3*a,b*). The Bayes factor analysis comparing treatment groups for both maternal and paternal body sizes revealed significant differences between treatment groups, with treatment groups that experienced late constant conditions generally having the largest body sizes (figure 4*a,b*).

### (c) Effect of thermal treatments on spawning success

We conducted 174 breedings in total, and of those, females spawned (viable and/or non-viable) in 132 (75.9% of trials). The Bayesian mixed model for spawning success revealed a relatively strong, positive interaction between early and late thermal treatments, such that experiencing the same temperature regime during both life-stages strongly enhanced spawning success (figure 3*c*). Further, there was a negative influence of early fluctuating temperature on spawning success (figure 3*c*). Paternal length also had a modest positive effect on spawning success (figure 3*c*). The effects of week of the experiment, family lineage and exposure to late fluctuating temperatures on spawning success were negligible (figure 3*c*). The Bayes factor analysis comparing treatment groups for spawning success revealed differences between mean values for the four treatment groups, such that CC and FF groups had the highest spawning success (figure 4*c*).

### (d) Effect of thermal treatments on female reproduction: fecundity

The number of viable eggs produced across trials ranged from 0 to 389. The Bayesian mixed model for fecundity revealed a negative influence of late fluctuating temperatures on fecundity (figure 4*d*). At the same time, there was a non-negligible, positive influence of early fluctuating temperatures on fecundity, albeit with moderate certainty (95% UIs overlap zero, but 50% UIs do not; figure 3*d*). There was a modest positive effect of week on fecundity, but maternal length was of negligible influence (figure 3*d*). There were differences in fecundity between treatment groups, based on a Bayes factor analysis, such that the FC and CC groups had the highest fecundity respectively (figure 4*d*).

### (e) Effect of thermal treatments on female reproduction: yolk volumes

We estimated yolk volumes for a total of 1194 eggs. Volumes ranged from 6.2 × 10^−2^ to 2.1 × 10^−1^ mm^3^. The Bayesian mixed model suggested yolk volumes were relatively strongly and negatively affected by late fluctuating temperatures (figure 3*e*). There was also a significant negative interaction between early and late fluctuating temperatures, that resulted in an additively negative impact on eggs from parents in FF treatments (figure 3*e*). Maternal length had a relatively weak negative influence on yolk volumes (figure 3*e*). The week of breeding had a positive effect on yolk volumes, and there were differences between family lineages (figure 3*e*). There were differences in yolk volumes between treatment groups, based on a Bayes factor analysis, such that CC and FC groups had the highest yolk volumes, and the FF group had appreciably lower yolk volumes (figure 4*e*).

### (f) Effect of thermal treatments on male reproduction: sperm concentration

We attempted to collect sperm samples from 36 individuals but were only able to collect 23 samples. We experienced difficulty collecting sperm from 12 males (i.e. were unable to collect sperm by the third attempt) and did not proceed with collection to prevent undue stress or mortality to the individuals. One sample was removed before spectrophotometry due to contamination with faeces.

The Bayesian linear model suggested paternal mass had a comparatively strong and positive effect on sperm concentration, but neither early nor late thermal treatment affected sperm concentration (figure 3*f*). Computed Bayes factors favoured the null hypothesis (i.e. that all treatment groups had the same sperm concentration, BF_10_ < 1 for all groups; figure 4*f*).

## 4. Discussion

Developmental plasticity and reversible acclimation may act alone or in tandem to shape the phenotypes of organisms in variable environments, but their contributions to variation in reproduction under ecologically relevant thermal variability are largely unknown. Here, we applied simulated diel thermal variability spanning early and late ontogenetic periods, with the goal of investigating the singular and interactive effects of developmental plasticity and reversible acclimation on reproductive traits in zebrafish. We found the ability of pairs to spawn was enhanced when their late ontogenetic environment matched that of early development. We also found complex interactions between early and late thermal experiences that ultimately shaped the fecundity-egg size relationship; early developmental exposure to thermal variability enhanced fecundity while concomitantly decreasing egg size, whereas late acclimation to thermal variability represented a constraint on both. Last, temperature did not exert direct effects on male fertility, but thermal variability's negative influence on male body sizes, the realized effect was a reduction in sperm quality. Overall, our results indicate that experiencing thermal variability largely led to decreases in reproductive metrics, but there was evidence to suggest plasticity significantly altered these effects.

Among the reproductive traits studied, the only one that exhibited clear beneficial acclimation was spawning success. Fish reared in the same environment throughout life showed greater spawning success, and notably, this positive effect still occurred regardless of whether the thermal regime was constant or variable. Interestingly, although male body size is expected to be a significant contributor to mating success [45], we detected only a minute positive effect of paternal length, which was dwarfed by that of thermal beneficial acclimation. It is possible that this beneficial acclimation of spawning success is due to individual mating preferences acquired during early development [51]. In this scenario, desirable phenotypes, including appearance, chemical signalling and behaviour, may have been conditioned by the early ontogenetic environment in anticipation of maturity [38,51]. This scenario would be both plausible and possibly adaptive if the temperatures experienced during early ontogeny act as reliable indicators of future breeding conditions, to the benefit of parental fitness [52]. Alternatively, it is possible that beneficial acclimation of performance traits such as aerobic capacity or swimming speed indirectly influenced males' chance of success during courtship, as others have found [53]. Although tests of these two possibilities were beyond the scope of this study, future behavioural observation or metabolism studies may elucidate the mechanisms behind beneficial acclimation of spawning success.

By contrast to spawning success, male fertility did not appear to exhibit beneficial acclimation. Instead, there was a significant detrimental effect of male body sizes on sperm quality [42], which was associated with thermal variability's negative impact on male body size during late ontogeny through acclimation. Other authors have likewise found developmental rearing temperatures do not impact fish sperm counts [54], which is unsurprising given that spermatozoa are regenerated daily in sexually mature fish [55]. Consequently, it would be prudent to consider both the timing- and sex-specific effects of temperature on body sizes of male fish going forward, given the cascading impact that temperature-driven changes in body size have on male fertility.

In response to variable temperature regimes, we detected evidence of plastic life-history trade-offs in fecundity and egg provisioning of females owing to developmental plasticity. Our results suggested that exposure to thermal variability during the first 30 days of development had a lasting, enhancing effect on fecundity, concomitant with a negative effect on yolk volumes when fish were exposed for their entire lives. Interestingly, these results are contrary to existing theoretical predictions and some empirical studies, which often support the expectation that variable or stressful conditions should lead to decreased fecundity and larger eggs [56–58].

However, these expectations of fecundity–egg provisioning relationships often assume that ‘bigger is better’—or, specifically, that well-allocated offspring are advantaged in unideal environments [58]. For conditions under which smaller offspring are favoured, this assumption is violated [59–63]. Under the thermally variable regime used in our study, it is possible that smaller offspring may ultimately experience metabolic advantages, especially given the lower oxygen solubility and higher energetic demands associated with increases in water temperature [59]. Further, in zebrafish, both fertilization and hatching success are significantly lower for larger eggs at hot constant temperatures (30°C) [40], suggesting a reduction in egg size may have adaptive benefits under diel thermal variability that crosses hot temperature thresholds. However, further experiments investigating fertilization rates and offspring survival, which were not studied herein, are needed to determine whether this unexpected life-history trade-off is indeed beneficial for parental fitness.

By contrast to developmental plasticity to thermal variability, acclimation had a negative effect on both fecundity and yolk volumes. It is likely that these effects are facultative (i.e. a direct and negative consequence of chronically experiencing suboptimal temperatures [64]). Given the metabolic demands required to cope with temperature fluctuations beyond thermal optima, fish exposed to the fluctuating regime for the latter portion of their lives likely expended greater portions of their energy budget toward somatic maintenance [65]. As a result, these fish had fewer total resources available to invest in reproduction [66]. This suggestion is supported by the fact that negative effects of fluctuating temperatures later in life were found on female body sizes, fecundity and yolk volumes alike, signalling a constraint. Overall, it is important to recognize that female fish reared in fluctuating treatments during late ontogeny still had appreciably lower total reproductive output than those reared in optimal conditions. Although developmental plasticity slightly enhanced fecundity, total compensation was not achieved; multiple generations of plastic changes or evolution may be needed to optimize reproductive output under increased thermal variability [67,68].

A notable, though unintended, feature of this study is that our constant temperature treatment was somewhat thermally variable. Thus, our results more closely represent a comparison between regimes with low versus high thermal variability. Interestingly, we note that estimates for spawning success, fecundity, yolk volume and sperm concentrations in our lifetime constant treatment group were similar to previously reported results in wild-type zebrafish held in explicitly optimal constant thermal conditions [69–71]. These results suggest that small fluctuations in temperature may have minor impacts, but the magnitude of response is dependent on the magnitude of variability, as others have found [20,22,64]. Ultimately, as the climate changes, we must address both thermal means and magnitudes of variability to accurately predict organismal responses [22,72].

It was beyond the scope of this study to investigate growth rates, age at maturity and reproductive lifespan of both parents and offspring, but these measures play important roles in the suite of connected life-history characteristics that can vary in response to environmental conditions, and collectively shape parental fitness [32]. For example, recent evidence suggests that temperature itself can modify the onset of sexual maturity independently of temperature-induced changes in somatic growth [73], and that temperature differentially influences lifespan and fecundity [74]. Future experiments examining fitness explicitly—including the onset of sexual maturity, breeding successes or failures, and subsequent offspring survival—will unequivocally address if and how developmental plasticity and acclimation influence fitness through life-history trade-offs.

The world is becoming both warmer, on average, as well as more thermally variable [75]. In this study, we show that thermal variability results in myriad changes to fundamental reproductive traits in zebrafish and that these effects are a result of a complex interplay between developmental plasticity and reversible acclimation. Our results support a significant role for developmental plasticity in the alteration of life history and awakened support for the beneficial acclimation hypothesis under ecologically realistic thermal regimes. Further tests into the adaptive value of these plastic changes will benefit our understanding of organismal performance under changing climatic conditions.

## Data Availability

Original data and code used in analyses are available via Dryad Digital Repository: https://doi.org/10.5061/dryad.7h44j0zx1 [50]. Electronic supplementary material including detailed methods, parameter estimates from Bayesian models, Bayes factor analyses results, visual model checking, and supplemental results on non-viable egg production are available at Figshare [76].
